# A Comprehensive Review of the Role of Stem Cells in Neuroregeneration: Potential Therapies for Neurological Disorders

**DOI:** 10.7759/cureus.67506

**Published:** 2024-08-22

**Authors:** Navanath Deokate, Sourya Acharya, Rajvardhan Patil, Suhail M Shaikh, Vineet Karwa

**Affiliations:** 1 Internal Medicine, Jawaharlal Nehru Medical College, Datta Meghe Institute of Higher Education & Research, Wardha, IND

**Keywords:** clinical trials, cell differentiation, therapeutic potential, neurological disorders, neuroregeneration, stem cells

## Abstract

Stem cell research has emerged as a groundbreaking field with significant potential for advancing neuroregeneration and neurological disorder treatment. Neurological conditions such as Alzheimer's disease, Parkinson's disease, stroke, and spinal cord injuries pose severe challenges due to their impact on quality of life and the limited efficacy of current treatments, which primarily focus on symptom management rather than addressing the underlying damage. Neuroregeneration, the process of repairing and restoring damaged neural tissues, is crucial for improving patient outcomes, given the central nervous system's limited intrinsic repair capacity. Stem cells offer a promising solution due to their ability to self-renew and differentiate into various neural cell types, providing opportunities for innovative therapies. This review provides a comprehensive analysis of the role of stem cells in neuroregeneration, exploring different types of stem cells, including embryonic stem cells (ESCs), induced pluripotent stem cells (iPSCs), and adult stem cells, and their mechanisms of action in neural repair. It examines current clinical trials and translational research efforts, highlighting successes and ongoing challenges such as ethical considerations, immunogenicity, and technical limitations. The review also discusses future directions in stem cell research, including advancements in gene editing, tissue engineering, and personalized medicine. By addressing these aspects, the review aims to offer a thorough understanding of the potential and challenges of stem cell-based therapies, contributing to the development of effective treatments for neurological disorders and ultimately enhancing patient quality of life.

## Introduction and background

Neurological disorders encompass various conditions affecting the brain, spinal cord, and peripheral nerves. These disorders, including Alzheimer’s disease (AD), Parkinson’s disease (PD), stroke, spinal cord injuries (SCIs), and multiple sclerosis (MS), present significant challenges globally, affecting millions of individuals and imposing a profound impact on their quality of life, functional independence, and mortality rates [1]. The World Health Organization (WHO) highlights neurological disorders as leading causes of disability-adjusted life years (DALYs), contributing substantially to global morbidity and mortality. The socioeconomic burden of these disorders is immense, with substantial healthcare costs, loss of productivity, and the need for long-term care and support [2]. Patients often endure progressive and debilitating symptoms, such as cognitive decline, motor dysfunction, sensory impairments, and psychological disturbances. Despite advances in medical research and clinical care, effective treatments remain limited, primarily focusing on symptom management rather than curative or regenerative approaches [3].

Neuroregeneration, the process of repairing, replacing, or regenerating damaged neural tissues, is essential for addressing the underlying causes of neurological disorders. The central nervous system (CNS) has a limited capacity for self-repair, making neuroregeneration a critical area of interest in neuroscience and regenerative medicine [4]. Successful neuroregeneration could potentially restore lost functions, improve clinical outcomes, and enhance the quality of life for individuals with neurological conditions. Advances in understanding the molecular and cellular mechanisms of neuroregeneration have opened new avenues for therapeutic interventions, including promoting endogenous repair processes, harnessing the potential of stem cells, and developing novel biomaterials and bioengineering approaches. These strategies aim to create an environment conducive to neural repair and functional recovery [5].

Stem cell research has emerged as a promising frontier in neuroregeneration, offering potential solutions for various neurological disorders. Stem cells possess unique properties, including the ability to self-renew and differentiate into various cell types, making them ideal candidates for regenerative therapies [6]. Different types of stem cells, such as embryonic stem cells (ESCs), induced pluripotent stem cells (iPSCs), and adult stem cells (e.g., neural stem cells, or NSCs; mesenchymal stem cells, or MSCs), have been extensively studied for their therapeutic potential in neuroregeneration. Research focuses on elucidating how stem cells contribute to neural repair, enhancing their efficacy and safety, and translating preclinical findings into clinical applications. Significant progress has been made in preclinical studies, demonstrating the potential of stem cells to differentiate into neural cells, promote neuroprotection, modulate the immune response, and support axonal growth and synaptic plasticity. Clinical trials are ongoing to assess the safety and efficacy of stem cell-based therapies in patients with various neurological conditions, with early results showing promise [7].

This comprehensive review aims to provide an in-depth analysis of the role of stem cells in neuroregeneration and their potential as therapeutic agents for neurological disorders. The objectives of this review are to elucidate the different types of stem cells and their unique properties relevant to neuroregeneration, explore the mechanisms by which stem cells contribute to neural repair and functional recovery, examine the current state of stem cell-based therapies for specific neurological disorders, including clinical trials and translational research, identify the challenges and limitations associated with stem cell therapies, such as ethical, technical, and safety concerns, and discuss future directions and innovations in the field, highlighting emerging technologies and potential areas for further research. By addressing these objectives, this review seeks to provide a comprehensive understanding of the potential and challenges in relation to stem cell-based therapies in neuroregeneration, contributing to the advancement of effective treatments for neurological disorders.

## Review

Types of stem cells

**Figure 1:**
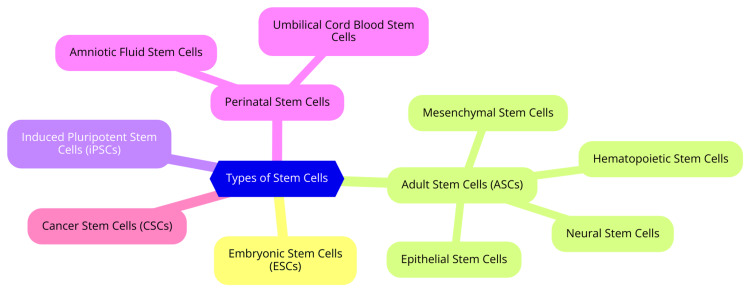
Types of stem cells Image credit: Dr Navanath Deokate

Stem cells are classified into various types based on their origin and potential, each offering distinct properties and applications in neuroregenerative medicine. This overview specifically examines embryonic stem cells, induced pluripotent stem cells, and adult stem cells, detailing their characteristics, applications, and limitations [8]. ESCs originate from the inner cell mass of blastocysts. They are categorized as pluripotent, meaning they can differentiate into any cell type in the body, including neurons and glial cells. This remarkable capability positions them as potent candidates for neuroregenerative therapies, potentially replacing damaged or lost neural cells in conditions like spinal cord injuries, Parkinson's disease, and other neurodegenerative disorders [9]. However, the use of ESCs raises significant ethical concerns due to the need to destroy embryos for their derivation. This ethical dilemma has resulted in stringent regulations and ongoing debates about the moral implications of employing human embryos in research. Additionally, there are challenges related to immune rejection when ESCs are transplanted into patients, as they are often perceived as foreign tissue by the immune system [10]. Induced pluripotent stem cells are generated by reprogramming adult somatic cells to revert them to a pluripotent state, mimicking the characteristics of ESCs. This groundbreaking technique, first successfully demonstrated in 2006, allows for creating patient-specific stem cells without the ethical concerns associated with ESCs [7]. iPSCs hold tremendous promise in neuroregenerative medicine as they can differentiate into various types of neural cells for both research and therapeutic applications. They are particularly valuable for modeling neurological diseases, conducting drug testing, and potentially providing autologous cells for transplantation, thus mitigating the risk of immune rejection [7]. Adult stem cells are present in various tissues and generally exhibit multipotent characteristics, meaning they can differentiate into a limited range of cell types specific to their tissue of origin. Key types include neural stem cells, which reside in specific brain and spinal cord regions. NSCs can generate neurons, astrocytes, and oligodendrocytes, playing a critical role in brain repair and regeneration following injury or neurodegenerative diseases. However, their limited availability and challenges associated with in vitro expansion pose significant hurdles for therapeutic applications [11]. Mesenchymal stem cells, found in tissues such as bone marrow and adipose tissue, are notable for their capacity to differentiate into multiple cell types, including osteoblasts and chondrocytes. In neuroregeneration, MSCs secrete neuroprotective factors and modulate inflammation, thereby supporting the repair of damaged neural tissue. Nonetheless, their differentiation potential is more restricted than ESCs and iPSCs [11]. Hematopoietic stem cells (HSCs) generate all types of blood cells, primarily in the bone marrow. While their primary function is hematopoiesis, research has explored their potential in treating neurological disorders through immune modulation and neuroprotection mechanisms. The main limitation of HSCs lies in their constrained differentiation capacity, primarily focusing on blood cell lineages [11]. Types of stem cells are shown in Figure 1.

Mechanisms of neuroregeneration

Stem cells can differentiate into various neural cell types, making them a promising tool for neuroregeneration. When transplanted into damaged neural tissues, stem cells can replace lost neurons, astrocytes, and oligodendrocytes, potentially restoring lost functions and improving neurological outcomes. The newly differentiated neurons can establish synaptic connections with existing neural circuits, facilitating functional integration and recovery [12]. In addition to direct cell replacement, stem cells contribute to neuroregeneration through neuroprotective mechanisms. They secrete various neurotrophic factors that promote the survival of existing neurons, reduce apoptosis, and enhance neuronal resilience to injury. Furthermore, stem cells can mitigate oxidative stress, a common feature in many neurological disorders, by producing antioxidants and modulating cellular pathways that protect neurons from damage [13]. The immune system plays a crucial role in the pathogenesis and progression of many neurological disorders. Stem cells can modulate the immune response in the nervous system, reducing inflammation, which is often detrimental to neural recovery. They secrete cytokines and other signaling molecules that influence the activity of microglia (the resident immune cells of the CNS), encouraging them to adopt a more neuroprotective phenotype, thereby promoting a conducive environment for regeneration [14]. Stem cells also contribute to neuroregeneration through paracrine effects and trophic support. They release neurotrophic factors (e.g., brain-derived neurotrophic factor, or BDNF; nerve growth factor, or NGF; glial cell-derived neurotrophic factor, or GDNF) that support neuronal survival, growth, and differentiation. These factors enhance the repair processes in damaged neural tissues. Moreover, paracrine signaling from stem cells to surrounding cells can stimulate endogenous repair mechanisms, promoting the regeneration of neural tissues [15]. Finally, stem cells can enhance axonal growth and synaptic plasticity, crucial for restoring connectivity and function in the nervous system. They promote axonal regeneration by enhancing the intrinsic growth capacity of neurons, facilitating axonal regrowth after injury. Additionally, stem cell-derived neurons can contribute to synaptic plasticity, the ability of synapses to strengthen or weaken over time, which is essential for learning, memory, and recovery of function following neurological insults [16].

Stem cell therapies for specific neurological disorders

Stem cell therapies hold significant promise for treating various neurological disorders, including neurodegenerative diseases, spinal cord injuries, stroke, traumatic brain injuries, multiple sclerosis, and Huntington's disease (HD) [17]. In the realm of neurodegenerative diseases, Alzheimer's disease is a prime target for stem cell therapies. Extracellular vesicles derived from bone marrow mesenchymal stromal cells (BM-MSC-EVs) and human adipose-derived stromal/stem cells (hASCs) have shown benefits in reducing Aβ42 deposits, increasing neuronal survival, and improving memory and learning abilities [18]. Promising results from stem cell therapies have also been seen in Parkinson's disease. iPSCs allow for accurate PD modeling, are patient specific (minimizing the risk of immune rejection), and have not been found to form tumors in the brain in long-term observations. Animal models have demonstrated increased dopaminergic neurons in the midbrain, with mature neurons extending dense neurites into the host's striatum, resulting in improved movement [18]. Amyotrophic lateral sclerosis (ALS) has benefited from MSC therapies, which have shown promise in slowing ALS progression and demonstrating early signs of efficacy. Bone marrow-derived MSCs induced to secrete neurotrophic factors increased cerebrospinal fluid neurotrophic factors, with all cell transplantations proving safe [18]. Stem cell therapies have also shown potential in treating spinal cord injuries. These therapies can replace lost neurons, promote axonal growth, and modulate the immune response to create a more favorable environment for regeneration [19]. Multiple sclerosis (MS) has seen promising results from MSC therapy, which has been more potent than immunomodulatory drugs in preventing excessive glial scarring in the central nervous system. This therapy has induced significant recalibration of pro-inflammatory and immunoregulatory components of the immune system [20]. HD has also benefited from MSC therapies, which have significantly impacted motor improvements, stimulating endogenous neurogenesis, reducing neuronal loss, and decreasing Huntingtin aggregation [21]. While these therapies show promise, further research is needed to optimize their efficacy and ensure long-term safety in human patients. Ongoing clinical trials and continued advancements in stem cell technology hold great hope for the future of regenerative medicine in treating neurological disorders [22]. A summary of stem cell therapies for specific neurological disorders is shown in Table 1.

**Table 1:** Stem cell therapies for specific neurological disorders

Neurological Disorder	Type of Stem Cells Used	Mechanisms	Key Findings
Alzheimer's disease	Bone marrow mesenchymal stromal cells, human adipose-derived stromal/stem cells	Reducing Aβ42 deposits, increasing neuronal survival	Improved memory and learning abilities
Parkinson's disease	Induced pluripotent stem cells	Differentiation into dopaminergic neurons, enhancing midbrain function	Increased dopaminergic neurons, improved movement
Amyotrophic lateral sclerosis (ALS)	Mesenchymal stem cells	Secreting neurotrophic factors, increasing cerebrospinal fluid neurotrophic factors	Slowed ALS progression, early signs of efficacy
Spinal cord injury	Neural stem cells, mesenchymal stem cells	Replacing lost neurons, promoting axonal growth, modulating immune response	Functional improvements, potential for significant motor function recovery
Multiple sclerosis	Mesenchymal stem cells	Preventing glial scarring, recalibrating immune components	Reduced disease activity, improved quality of life
Huntington's disease	Mesenchymal stem cells	Stimulating endogenous neurogenesis, reducing neuronal loss	Motor improvements, decreased Huntingtin aggregation

Clinical trials and translational research

Recent advancements in stem cell therapy have spurred numerous clinical trials to address neurodegenerative diseases. This overview highlights completed and ongoing trials, key findings, challenges in translating preclinical results to clinical settings, and notable case studies [23]. Stem cell therapies have been extensively studied across a spectrum of neurodegenerative diseases, including Alzheimer's, Parkinson's, and MS. A comprehensive review identified 33 trials utilizing NSCs and eight trials employing differentiated neural cells. Furthermore, over 315 registered clinical trials targeted brain diseases, with MSCs and HSCs playing significant roles. Notably, approximately 102 trials focused specifically on MSCs, primarily for conditions such as stroke, SCI, and MS [24]. Early-phase trials have demonstrated promising safety profiles for MSCs and NSCs, with some indications of efficacy in slowing disease progression or improving symptoms. For instance, MSC therapy has shown potential benefits in ALS and HD by reducing disease markers and promoting neurogenesis. Stem cells exert therapeutic effects by directly replacing damaged cells or providing neuroprotective support via paracrine signaling, enhancing endogenous repair mechanisms [18]. Despite encouraging results, translating preclinical findings to clinical settings presents significant challenges. Many trials lack standardized methodologies, hindering comparative analysis of outcomes. The high costs of conducting large-scale, randomized controlled trials pose feasibility challenges, particularly for rare diseases. Additionally, navigating the regulatory landscape for stem cell therapies is complex and can impact the speed of clinical application [25]. Noteworthy case studies include a phase I clinical trial using umbilical cord MSCs for Parkinson's disease, which reported improved motor function among patients. Trials involving the transplantation of adult neural stem/progenitor cells for spinal cord injury have also shown functional improvements, suggesting potential for significant motor function recovery. HSC therapy has demonstrated efficacy in modulating immune responses in MS patients, reducing disease activity and improving quality of life [18]. The field of stem cell therapy for neurodegenerative diseases is rapidly advancing with numerous ongoing clinical trials. While notable successes and promising early results remain, substantial challenges are found in translating these findings into widespread clinical practice. Continued research and well-designed trials are crucial to establish the safety and efficacy of these innovative therapies [17].

Challenges and limitations

Applying stem cell therapies in neuroregeneration holds significant promise for treating neurological disorders; however, several challenges and limitations must be addressed to ensure their safe and effective use. One of the foremost challenges lies in the ethical and regulatory considerations surrounding stem cell research and therapy [26]. The use of embryonic stem cells raises ethical concerns due to the destruction of embryos during their derivation, leading to a preference for adult stem cells or iPSCs, which do not involve such ethical dilemmas. Additionally, the regulatory landscape for stem cell therapies is complex. It varies by country, making navigating these regulations challenging for researchers and clinicians and potentially delaying translating promising therapies from laboratory settings to clinical applications [26]. Another critical challenge is the immunogenicity of transplanted stem cells. When stem cells are derived from a donor (allogeneic cells), they can be recognized as foreign by the recipient's immune system, leading to rejection [27]. This risk is notably lower with autologous cells derived from the patient. Researchers are exploring various strategies to mitigate rejection, such as using immunosuppressive agents, genetically modifying stem cells to express anti-inflammatory factors, and developing personalized iPSCs tailored to individual patients' immune profiles [27].

Tumorigenicity is also a significant concern in stem cell therapies, particularly pluripotent stem cells. Undifferentiated stem cells have the potential to proliferate uncontrollably, leading to the formation of teratomas or other tumors. This risk is particularly pertinent with iPSCs and ESCs, which can differentiate into any cell type. To address this issue, ongoing research focuses on ensuring the complete differentiation of stem cells before transplantation and implementing rigorous monitoring for any signs of tumor development post-treatment [28]. Technical and manufacturing challenges further complicate the application of stem cell therapies. The lack of standardized protocols for isolating, expanding, and differentiating stem cells can lead to variability in clinical outcomes. Additionally, scaling up the production of high-quality stem cells for clinical use poses significant hurdles. Ensuring that stem cells maintain their therapeutic properties during large-scale production is crucial for successful patient application [29]. Finally, long-term safety and efficacy concerns remain a significant barrier to the widespread adoption of stem cell therapies. Many studies focus on short-term outcomes, creating a gap in understanding the long-term effects of stem cell transplantation. Longitudinal studies are essential to assess the durability of therapeutic benefits and identify any delayed adverse effects. Moreover, individual patient responses to stem cell therapy can vary widely due to genetic, environmental, and health factors, complicating the assessment of efficacy and safety across diverse populations [30].

Future directions and innovations

Recent advancements in biotechnology are revolutionizing various fields, particularly in medicine. One of the most significant breakthroughs has been in gene editing, especially with the advent of CRISPR (Clustered Regularly Interspaced Short Palindromic Repeats) technology. This revolutionary tool enables precise genome modifications, allowing researchers to target specific genes for therapeutic purposes. Recent developments include next-generation CRISPR tools, such as base editing and prime editing, which facilitate targeted modifications without introducing double-strand breaks (DSBs) [31]. These innovations reduce potential off-target effects and improve the safety profiles of gene therapies. The first CRISPR-based human therapy was approved in late 2023, marking a significant milestone in clinical applications. Ongoing research is focused on enhancing delivery methods for CRISPR systems, with non-viral vectors being explored to improve targeting and minimize immunogenic responses [32]. Another exciting area of innovation is 3D bioprinting and tissue engineering. This technology transforms the field by enabling the fabrication of complex tissue structures for transplantation and drug testing. 3D bioprinting allows for customizable scaffolds that mimic the extracellular matrix, supporting cell growth and differentiation [33]. When combined with stem cell therapy, bioprinting can enhance the regeneration of damaged tissues, offering potential solutions for conditions such as spinal cord injuries and heart disease. Integrating bioprinting and stem cell technology holds promise for developing functional tissues that can be used in regenerative medicine [34]. Developing biomaterials and scaffolds is crucial for effective tissue engineering and regenerative medicine. Innovations in this area include smart biomaterials that can respond to environmental stimuli, such as pH or temperature, allowing for the controlled release of therapeutic agents and improved integration with biological tissues. Additionally, new biodegradable materials are being developed to support tissue regeneration while gradually being absorbed by the body, reducing the need for surgical removal. These advancements in biomaterials enhance the potential for successful tissue engineering applications [35]. Personalized medicine is gaining traction as a critical approach in modern healthcare, focusing on tailoring treatments to individual patient profiles. Advances in genomic profiling technologies allow for the identification specific genetic mutations in patients, enabling targeted therapies that are more effective and have fewer side effects. Furthermore, integrating patient-specific data with treatment protocols can optimize therapeutic outcomes, particularly in complex diseases like cancer. This shift towards personalized medicine represents a significant advancement in how medical treatments are approached, moving away from the one-size-fits-all model [36].

The future of treatment modalities increasingly involves integrating various therapeutic approaches. For instance, combining stem cell therapies with neurorehabilitation techniques and pharmacological interventions can enhance recovery in neurological disorders. This multidisciplinary approach aims to maximize the regenerative potential of stem cells while providing supportive care [37]. By integrating gene editing, tissue engineering, and personalized medicine, future therapies may offer comprehensive solutions that address the underlying causes of diseases rather than merely managing symptoms. These innovations represent a significant leap toward more effective and personalized treatment options across various medical fields, particularly regenerative medicine and genetic disorders. Continued research and development are essential to overcome existing challenges and fully realize the potential of these technologies [38].

## Conclusions

In conclusion, stem cell research holds transformative potential for neuroregeneration and the treatment of neurological disorders. Stem cells offer unique advantages due to their ability to self-renew and differentiate into various neural cell types, presenting opportunities to address the limitations of conventional therapies. Advances in understanding the mechanisms of neuroregeneration, coupled with significant progress in preclinical and clinical research, underscore the promise of stem cell-based therapies in restoring neural functions and improving patient outcomes. However, the path forward is not without challenges, including ethical concerns, technical difficulties, and safety issues that need to be addressed to fully realize the potential of these therapies. Future research and innovations, such as advancements in gene editing, 3D bioprinting, and personalized medicine, are crucial for overcoming these hurdles and advancing the field. As we continue to explore and refine stem cell-based approaches, there is hope for more effective and durable treatments for neurological disorders, ultimately enhancing the quality of life of millions of individuals affected by these debilitating conditions.
